# The impact of digital health on hospitalization and mortality in heart failure patients in low- and middle-income countries: a systematic review and meta-analysis

**DOI:** 10.1093/ehjdh/ztag117

**Published:** 2026-07-22

**Authors:** Preston Nicely, Emily Eichstaedt, Charles Heald, Jack Simmons, Shiv Ishpujani, Stephen Clarkson

**Affiliations:** Department of Internal Medicine, The University of Alabama Birmingham, Medical Towers, MT 621, 1720 2nd Avenue South, Birmingham, AL 35294-4410, USA; Heersink School of Medicine, The University of Alabama Birmingham, 701 19th Street South, ALGEN 7, Birmingham, AL 35233, USA; Department of Internal Medicine, The University of Alabama Birmingham, Medical Towers, MT 621, 1720 2nd Avenue South, Birmingham, AL 35294-4410, USA; Department of Internal Medicine, The University of Alabama Birmingham, Medical Towers, MT 621, 1720 2nd Avenue South, Birmingham, AL 35294-4410, USA; Department of Internal Medicine, The University of Alabama Birmingham, Medical Towers, MT 621, 1720 2nd Avenue South, Birmingham, AL 35294-4410, USA; Division of Cardiovascular Disease, The University of Alabama Birmingham, Tinsley Harrison Tower, Suite 311, 1900 University Boulevard, Birmingham, AL 35233, USA

**Keywords:** Low- and middle-income countries, Digital health, mHealth, Heart failure, Monitoring, Telehealth

## Abstract

**Aim:**

Heart disease is the leading cause of death worldwide, and the growing prevalence of heart failure (HF) is a key contributor. Digital health (dHealth) modalities including mobile applications, telehealth, and wearables have been developed to improve HF care, though little is known about their impact in low- and middle-income countries (LMICs). The aim of this review is to identify studies from LMICs that evaluated dHealth interventions for HF and determine the impact of those interventions on hospitalization and mortality.

**Methods and results:**

Six databases were searched from 1 January 1990 to 11 June 2025. A total of 3524 articles were screened (Cohen’s κ = 69%). Included studies had a dHealth intervention, were a randomized controlled trial (RCT), occurred in a LMIC, and reported outcomes of interest. Risk of bias for RCTs was assessed using the Cochrane Risk of Bias 2 Tool. Fifteen RCTs (*n* = 4568) with studies in Brazil (33.3%), China (26.7%), and Thailand (13.3%) were included. Interventions included telemonitoring, mobile apps, and wearables. The population averaged 61.1 years, 31.4% female, and 45.7% New York Heart Association class III–IV. Random-effects meta-analysis showed dHealth reduced all-cause hospitalizations [risk ratio (RR) 0.73, 95% confidence interval (CI) 0.59–0.90], HF hospitalizations (RR 0.70, 95% CI 0.59–0.84), and all-cause mortality (RR 0.86, 95% CI 0.79–1.00); HF mortality was neutral (RR 0.64, 95% CI 0.37–1.11).

**Conclusion:**

Digital health in LMICs lowers hospitalizations and may reduce all-cause mortality, though the benefit for HF mortality remains uncertain. These findings support broader dHealth adoption in LMICs to address HF burden.

PROSPERO registration number: CRD420251079715

## Graphical Abstract

**Graphical Abstract:**
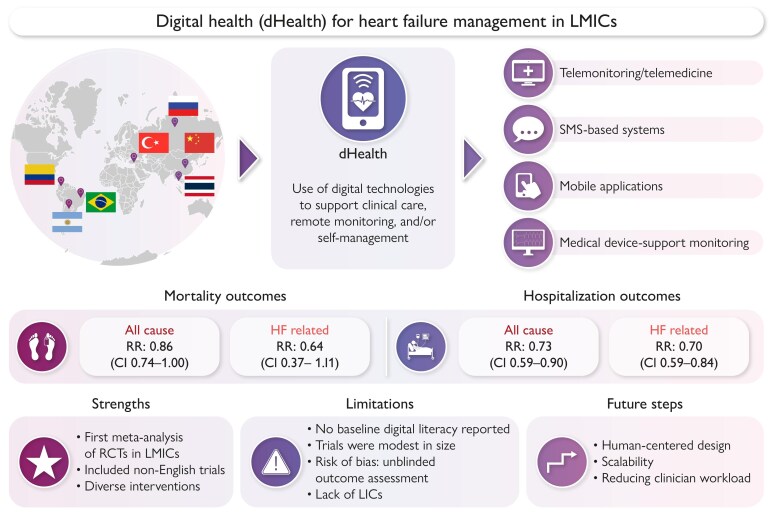

## Introduction

Heart failure (HF), defined as a clinical syndrome arising from structural or functional myocardial abnormalities that impair the heart’s ability to pump or fill adequately, is a growing public health concern with rising prevalence and high morbidity; specifically, the years lived with disability due to HF have increased across the globe from about 2.4 million in 1990 to over 5.3 million in 2021.^1^ Approximately 60 million people are living with HF globally, likely due to an ageing population, improvement in cardiac survival post major adverse cardiac event, and an increasing prevalence of cardiometabolic disease.^1,2^ Many low- and middle-income countries (LMICs) are undergoing a rapid epidemiological transition in which declining infectious-disease mortality has accelerated the rise of non-communicable diseases, including HF, as major contributors to morbidity and mortality.^3^ Although HF affects every region, its clinical and health-system burden is greatest in LMICs, which account for nearly 80% of global cardiovascular disease deaths and face higher rates of advanced presentation, hospitalization, and limited access to longitudinal care.^2,4^ In high-income countries (HICs), HF is typically due to chronic ischaemic heart disease as compared to LMICs, where non-ischaemic aetiologies like hypertension, rheumatic heart disease, and Chagas disease are more prevalent.^5^

The magnitude of HF in LMICs has made the search for scalable, cost-effective strategies a global priority. In this context, digital health (dHealth) has emerged as a promising approach to strengthening chronic disease management, including HF. Digital health is broadly defined as the use of digital technologies such as social media platforms, mobile phones, personal computers, internet-connected devices, and even artificial intelligence to interface with individual or community populations in support of clinical care, remote monitoring, and disease self-management.^6^

Common dHealth modalities include telemonitoring/telemedicine, mobile health (mHealth), health information technology, and wearable devices. These technologies align closely with the needs of HF management, as continuous symptom surveillance, early detection of decompensation, medication reminders, remote consultation, and structured patient education can all be delivered through digital platforms at relatively low marginal cost.^7,8^

Though the evidence is largely limited to HICs, telemonitoring, mobile application-based interventions, remote educational modules, and connected devices have demonstrated promising effects in several HF trials.^7,9^ The efficacy of dHealth in HICs is supported by robust infrastructure, stable network connectivity, and high digital literacy among patients and clinicians.^7^ However, the potential value of dHealth may be even greater in LMICs, where health systems face limited cardiology workforce capacity, fragmented referral pathways, geographic barriers, inconsistent access to follow-up care, and shortages of diagnostic infrastructure.^10,11^ The current evidence for dHealth interventions in LMICs for HF is fragmented and scarce, and a dedicated synthesis of dHealth interventions specifically within these populations is needed. Thus, the objective of this review is to evaluate the existing evidence on dHealth interventions for HF management in LMICs and assess their impact on hospitalization and mortality.

## Methods

### Study design

The aim of this systematic review was to evaluate dHealth interventions on HF hospitalization and mortality in LMICs. The eligibility criteria for the research question were defined *a priori* using the population, intervention, control, outcomes framework (see Supplementary material online, *Table S1*). Studies from 1 January 1990 to 11 June 2025 evaluating the use of dHealth for HF management in LMICs were included as limited literature on this topic existed prior to 1990. Management of HF was defined broadly and included remote symptom monitoring and reporting, pharmacologic intervention or titration, HF education, healthcare utilization, referral to cardiology, and linkage to cardiac rehab. Digital health interventions included, but were not limited to, mentions of mHealth, short messaging services (SMS), telemedicine/telemonitoring, the internet, artificial intelligence, and social media platforms. Studies focusing on the diagnosis of HF or the diagnosis and management of non-HF cardiac conditions were not included in this review, despite dHealth utilization. Supplementary material online, *Table S4* details specific inclusion and exclusion criteria regarding dHealth adapted from similar criterion in the literature,^12^ and Supplementary material online, *Table S5* provides specific descriptions of each study’s dHealth technology. Only studies with a randomized controlled trial (RCT) design were included. Case reports, retrospective cohort studies, case–control studies, case series, editorial/opinion pieces, and review articles were excluded. low- and middle-income countries were defined according to the 2024–2025 World Bank country income classification. This system categorizes economies annually based on gross national income per capita and assigns countries to four income groups: Low-income, lower-middle-income, upper-middle-income, and high-income.^13^ Outcomes of interest included HF hospitalizations, HF-related mortality, all-cause hospitalization, or all-cause mortality. Additional secondary outcomes including health-related quality of life, functional capacity, and self-management measures were extracted when available and synthesized qualitatively. However, they were not included in the quantitative meta-analysis. Studies that reported these later metrics alone without primary outcomes of interest were excluded.

### Search strategy

**Figure 1:**
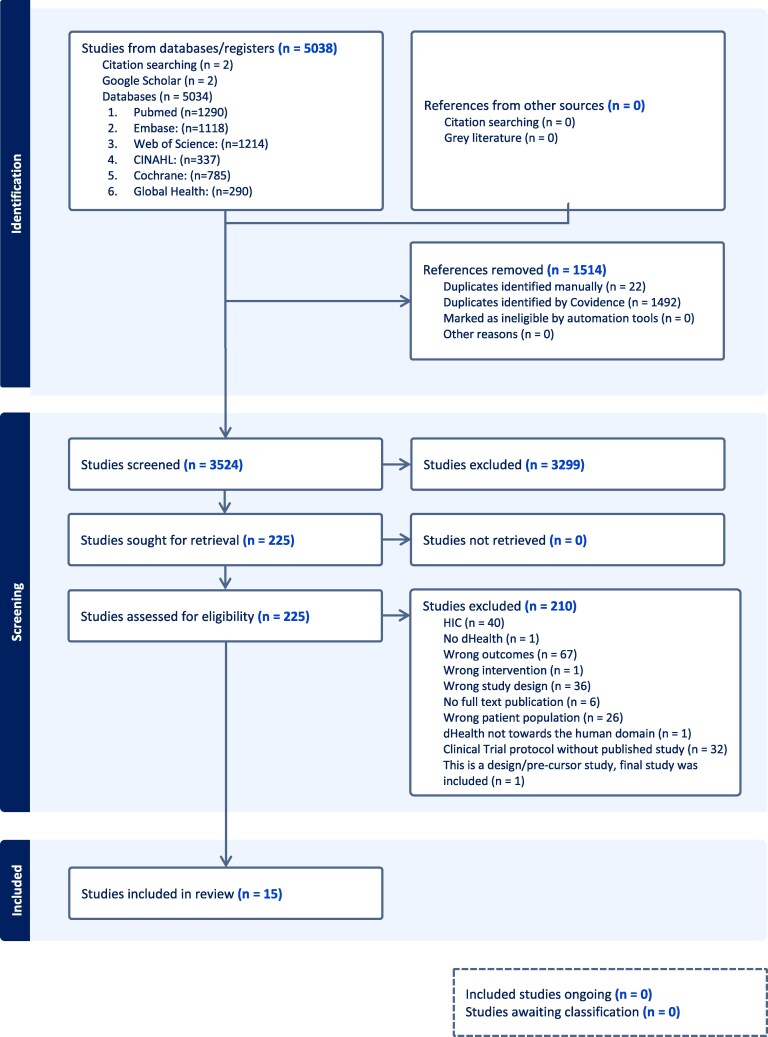
PRISMA diagram.

A search strategy was executed in accordance with the Cochrane Collaboration Guidelines with a single literature search conducted on 11 June 2025.^14^ Search terms were developed via literature review and guidance of a medical librarian. The syntax of the search terms was formatted using the polyglot search feature available through the Systematic Review Accelerator.^15^ Six electronic databases were systematically searched: PubMed, Embase, Global Health Database, CINAHL, Web of Science, and Cochrane. Grey literature was searched through targeted exploration of organizational reports, conference proceedings, clinical trial registries, and policy documents using structured keyword queries and manual screening of reference lists to identify unpublished or non-peer-reviewed evidence relevant to dHealth interventions in LMICs. Free text terms and standardized MeSH headings/subheadings in the context of Boolean operators and appropriate search term truncation were utilized to optimize sensitivity for relevant literature while minimizing excess search results (see Supplementary material online, *Table S2*). The search results were imported into Endnote™, and duplicate studies were removed.^16^ Studies not published in English were translated using an opensource Microsoft Translator application.^17^ The Preferred Reporting Items for Systematic Reviews and Meta-Analyses (PRISMA) study selection flow diagram is shown in *Figure 1*. The protocol for this systematic review was prospectively submitted on PROSPERO (registration No. CRD420251079715).

The search strategy was conducted under a broad PROSPERO-registered protocol designed to evaluate dHealth interventions for HF across multiple outcome domains and study designs. Following protocol registration, the research was further structured into three complementary analyses to address distinct research aims: (i) dHealth interventions for the diagnosis of HF in LMICs, (ii) dHealth interventions for objective clinical outcomes (hospitalization and mortality), and (iii) dHealth interventions for patient-centred and subjective outcomes, including health-related quality of life and composite endpoints, with Aim 2 encompassing the scope of this work. Accordingly, the inclusion and exclusion criteria for this analysis were refined and deviate from the PROSPERO protocol to align with Aim 2. The studies in this review were restricted to RCTs reporting discrete clinical endpoints (hospitalization and mortality) suitable for meta-analysis using risk ratios (RRs). Studies evaluating diagnostic applications, patient-reported outcomes, and biomarker-driven endpoints, without event-based data, were excluded from this analysis, but retained within the broader research protocol for analysis in companion work addressing Aims 1 and 3. Excluded studies and the rationale for their exclusion can be found in Supplementary material online, *Table S6*. Further details regarding differences in the PROSPERO protocol and the methodology of this review can be found in Supplementary material online, *Table S7*.

### Study selection

The titles and abstracts from the search were independently screened by three independent investigators (C.H., E.E., S.I.) using Covidence^TM^, a web-based systematic review software platform.^18^ The full-text articles of abstracts deemed to be potentially eligible were subsequently screened for inclusion using the same pre-specified criteria. A fourth reviewer (P.N.) adjudicated discrepancies between reviewers in the abstract and full-text screening phases.

### Data processing

A data extraction form was created on Covidence™. Extracted data items included first author name, study setting (e.g. country, world bank development index, funding source, sample size, publication year), participant characteristics (e.g. age, sex, race, key populations), study design (e.g. eligibility criteria, method of recruitment, loss to follow-up, risk of bias, study setting), intervention descriptions (frequency of interaction, dHealth modality), and outcomes. Data extraction was done independently by four separate reviewers (C.H., S.I., J.S., and E.E.) and then cross-checked by a fifth reviewer (P.N.). dHealth interventions were initially classified using a comprehensive framework encompassing multiple potential modalities, including telemonitoring/telemedicine, SMS-based interventions, mobile applications, device-supported monitoring, social media platforms, websites, artificial intelligence, digital vending systems, and other emerging technologies.^6^ However, among the included studies, interventions were ultimately represented by a subset of these categories as no studies were identified that utilized social media platforms, websites, artificial intelligence, or digital vending systems. For consistency, dHealth interventions were ultimately grouped into four predominant modalities observed across included studies: telemonitoring/telemedicine, SMS-based interventions, mobile application-based interventions, and device-supported monitoring. Individual dHealth modalities were defined according to the descriptions provided in their respective studies within the context of the scoping definition. The primary outcomes of interest included HF hospitalizations, HF-related mortality, all-cause hospitalization, or all-cause mortality. Heart failure hospitalizations and HF-related mortality were defined as hospitalization or death directly attributed to underlying HF or HF exacerbations and were explicitly stated as such in the source material.

### Data synthesis and risk of bias assessment

The Cochrane Risk of Bias Tool 2.0 was utilized to assess the strength of evidence and assess the risk of bias, which covers sequence generation, allocation concealment, binding, incomplete outcome data, and selective outcome reporting.^19^ Four researchers independently performed the assessment of quality and risk of bias in the individual studies and verified all assessments. Based on the initial literature review and perceived quality of evidence, a qualitative narrative synthesis of the data was performed. Data tables included study design features, study participant characteristics, descriptions of interventions, outcome results, risk of bias/methodological quality, outcomes, and results.

### Statistical analysis

Quantitative analyses were conducted in R (R Foundation for Statistical Computing) using the metafor and meta packages for effect estimation, heterogeneity testing, and creation of forest and funnel plots.^20^ Inter-observer agreement for study selection was assessed with Cohen’s κ.^21^ Characteristics of the study populations were summarized using standard descriptive statistics. Outcomes of interest included HF hospitalizations, all-cause hospitalizations, HF mortality, and all-cause mortality, which were extracted and analysed across all eligible RCTs in accordance with PRISMA guidelines. Meta-analysed pooled results for each hospitalization and mortality endpoint were generated using random-effects models (DerSimonian–Laird) with corresponding 95% confidence intervals (CIs), given the expected clinical and methodological heterogeneity across LMIC settings. For all efficacy endpoints, RRs served as the primary effect measure. Forest plots were constructed from the meta-analytic models, and statistical heterogeneity was quantified using the *I*^2^ statistic. A sensitivity analysis excluding studies with atypical designs or distinct patient populations was performed to evaluate the stability of pooled estimates. Subgroup analyses included intervention modality, geographic region, and study quality. Finally, a funnel plot with Egger’s test was generated to assess publication bias.

## Results

### Study selection

The systematic search identified 5038 records, of which 3524 unique records remained after duplicate removal. After title and abstract screening, 225 full texts were reviewed, and 15 RCTs met inclusion criteria (Cohen’s κ = 0.69). The most common reasons for exclusion included: study took place in a HIC (*n* = 40), wrong outcomes of interest (*n* = 24), wrong study design (*n* = 81), wrong patient population (*n* = 26), and incomplete study-clinical trial protocol without published study (*n* = 30). The PRISMA flow diagram, shown in *Figure 1*, provides an overview of the selection process.

### Study characteristics

**Figure 2:**
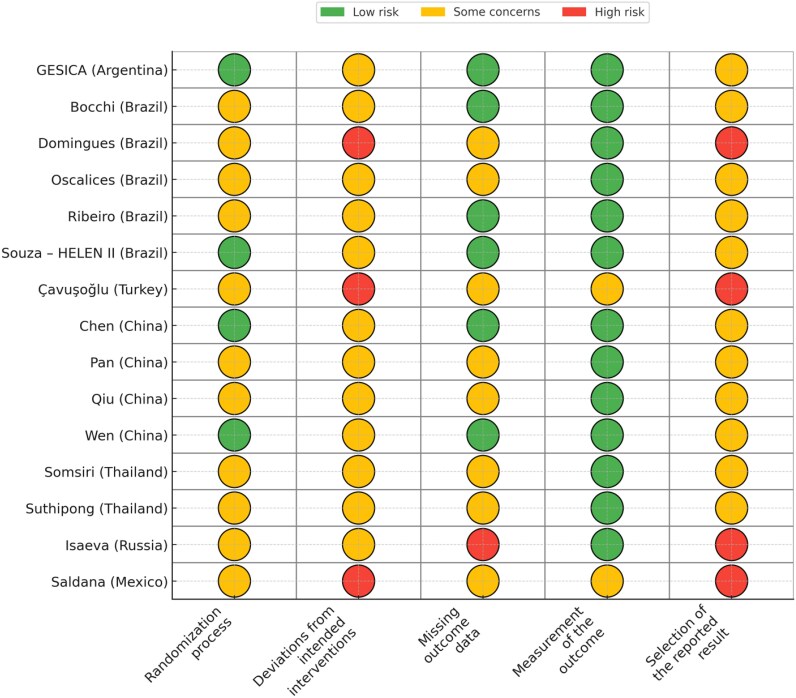
Risk of bias traffic plot of included studies.

The 15 included RCTs enrolled 4568 adults with HF across eight LMICs. *Table 1* details the characteristics of this study population. Studies were conducted primarily in Brazil,^22–26^ China,^27–30^ and Thailand,^31,32^ with additional trials from Turkey,^33^ Russia,^34^ Argentina,^35^ and Colombia,^36^ (*Figure 2*). Sample sizes ranged from 96 to 1518 participants, with follow-up durations from 2 to 12 months. Across trials, the mean age was 61.1 years, 31.4% were female, and 45.7% had HF with NYHA class III–IV symptoms at baseline. Most studies enrolled predominantly HFrEF populations with an average ejection fraction (EF) of 34%, though EF values were only reported in 8/15 studies, and Qiu and Gaoquin included mixed EF cohorts. Digital interventions were categorized into standardized modalities, including telemonitoring/telemedicine programmes,^22–26,33,35,36^ SMS-based systems,^27^ mobile application–based interventions,^28,29,31,32^ and device-supported monitoring systems.^30^ Control groups received standard HF care and routine clinic follow-up without structured digital support as defined within the context of each study.

**Table 1:** Demographics of included studies

Study (country)	Sample size	Mean age (years)	% Female	NYH A III–IV (%)	Intervention type	Intervention frequency	Mean follow-up duration (months)
Gesica (Argentina)	1518	65	29	49	1	Calls of variable duration (minutes), with the first 4 calls occurring every 2 weeks	16
Bocchi (Brazil)	350	51.5	31	38	1	Calls of variable duration every 2 weeks	∼30
Domingues (Brazil)	111	63	42	—	1	Calls of variable duration, weekly for 1st month, then q15 days for months 2–3 (8 calls total). First call was 7 days post-discharge	3
Oscalices (Brazil)	201	62.6	59	—	1	Calls ∼10 min long at 7 days and 30 days after discharge	3
Ribeiro (Brazil)	127	64	48	65	1	Twice weekly	6
Souza –HELEN II (Brazil)	252	62	37	56	1	10-min calls on day 10, 30, 60, and 120 post discharge	6
Çavuşoğlu (Turkey)	248	61	27	61	1	Calls of various duration at 1 month, 3 month, 6 months	6
Chen (China)	767	61	56	∼68	2	SMS group—received messages in the first 10 days of discharge and the reminder messages were repeated weekly for 1 month. STS group—received messages within the first 30 days after discharge	6
Pan (China)	96	68	48	100	3	Daily use with check ins at 1 month, 2 months, 3 months, and 6 months after discharge	6
Qiu (China)	133	54	30	68	3	3 days prior to discharge, download app, daily use with follow up at 3 months, 6 months, and 1 year	12
Gaoquin (China)	133	55	34	36	4	EKG routinely transmitted once in the morning, noon, and evening, plus phone calls at 1, 3, 6 months	6
Somsiri (Thailand)	145	61	28	43	3	Daily for 4 weeks, then every other day for last 2 weeks	2
Suthipon g (Thailand)	134	58	28	0	3	Daily	3
Isaeva (Russia)	213	69	53	—	1 + 3	Intervention 1: once or twice monthly Intervention 2: daily and q3 days	12
Achury Saldana (Colombia)	140	67	29	12	1	Daily	6 months

Intervention type: 1, telemonitoring/telemedicine; 2, SMS-based interventions; 3, mobile application–based interventions; 4, device-supported monitoring.

### Risk of bias

**Figure 3:**
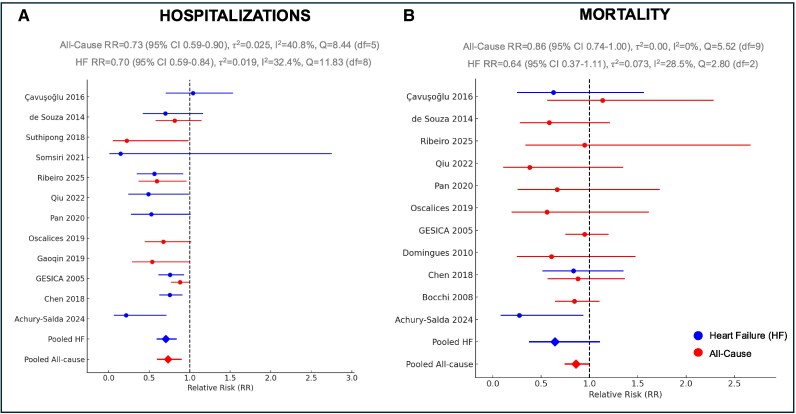
Forest plots of hospitalization (panel A) and mortality (panel B).

Risk of bias was assessed for all 15 RCTs using the Cochrane Risk of Bias 2 tool, across five domains: (i) bias arising from the randomization process; (ii) bias due to deviations from intended interventions; (iii) bias due to missing outcome data; (iv) bias in measurement of the outcome; and (v) bias in selection of the reported result. Overall, four trials were judged at low risk of bias, nine as having some concerns, and two as high risk of bias. A visual summary of these judgments is provided in the traffic-light plot (*Figure 3*) and in *Table 2*. Outcome definitions for hospitalizations and mortality were consistent across studies.

**Table 2:** Risk of bias summary

Study	Randomization	Deviations	Missing data	Outcome measurement	Selective reporting	Overall
GESICA (Argentina)	Low risk	Some concerns	Low risk	Low risk	Some concerns	Some concerns
Bocchi (Brazil)	Some concerns	Some concerns	Low risk	Low risk	Some concerns	Some concerns
Domingues (Brazil)	Some concerns	High risk	Some concerns	Low risk	High risk	High risk
Oscalices (Brazil)	Some concerns	Some concerns	Some concerns	Low risk	Some concerns	Some concerns
Ribeiro (Brazil)	Some concerns	Some concerns	Low risk	Low risk	Some concerns	Some concerns
Souza—HELEN II (Brazil)	Low risk	Some concerns	Low risk	Low risk	Some concerns	Some concerns
Çavuşoğlu (Turkey)	Some concerns	High risk	Some concerns	Some concerns	High risk	High risk
Chen (China)	Low risk	Some concerns	Low risk	Low risk	Some concerns	Some concerns
Pan (China)	Some concerns	Some concerns	Some concerns	Low risk	Some concerns	Some concerns
Qiu (China)	Some concerns	Some concerns	Some concerns	Low risk	Some concerns	Some concerns
Gaoquin (China)	Low risk	Some concerns	Low risk	Low risk	Some concerns	Some concerns
Somsiri (Thailand)	Some concerns	Some concerns	Some concerns	Low risk	Some concerns	Some concerns
Suthipong (Thailand)	Some concerns	Some concerns	Some concerns	Low risk	Some concerns	Some concerns
Isaeva (Russia)	Some concerns	Some concerns	High risk	Low risk	High risk	High risk
Achury-Saldana (Colombia)	Some concerns	High risk	Some concerns	Some concerns	High risk	High risk

### Primary Outcomes

**Figure 4:**
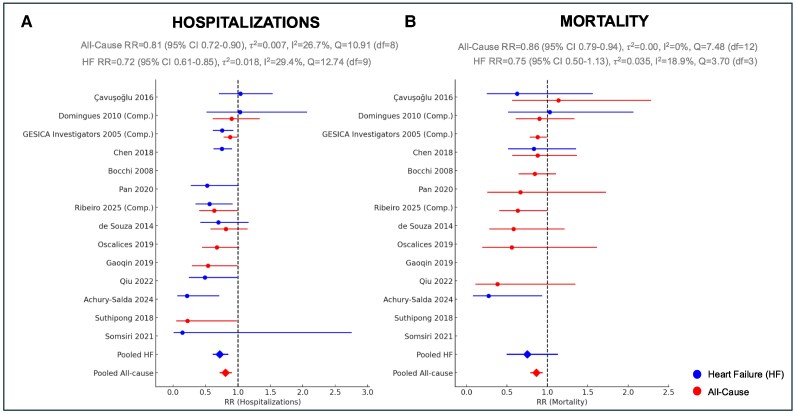
Geographic distribution of studies on heart failure in low- and middle-income countries.

dHealth interventions significantly reduced all-cause hospitalization (RR 0.73, 95% CI 0.59–0.90, *I*^2^ = 40.8%) per pooled estimates, *Figure 4*. Reductions were observed across multiple modalities, including telemonitoring/telemedicine^22–26,35^ and mobile application-based interventions.^28,29,31,32^ Among 13 studies reporting HF admission outcomes, interventions produced a significant reduction in HF hospitalization (RR 0.70, 95% CI 0.59–0.84, *I*^2^ = 32.4%). The strongest reductions were observed in Brazilian post-discharge nurse-led telemedicine programmes (de Souza: RR 0.55, 95% CI 0.38–0.79; Domingues: RR 0.62, 95% CI 0.44–0.87) and in China-based mobile application-based self-monitoring interventions (Pan: RR 0.58, 95% CI 0.41–0.82; Qiu: RR 0.46, 95% CI 0.28–0.76). Ten trials contributed to the mortality analysis.

Digital interventions were associated with lower all-cause mortality (RR 0.86, 95% CI 0.79–1.00, *I*^2^ = 0%). This effect was directionally consistent across studies, with mortality point estimates favouring dHealth in both large trials^35^ and smaller mobile application–based interventions.^28,29^ Heart failure mortality was reported in six studies and showed a nonsignificant trend towards improvement (RR 0.64, 95% CI 0.37–1.11, *I*^2^ = 28.5%).

### Secondary outcomes

In addition to the quantitative mortality and hospitalization outcomes, the studies included in this review reported various secondary outcomes from which qualitative assessments were synthesized. Quality-of-life improved across trials (*n* = 2) reporting Minnesota Living with Heart Failure Questionnaire (MLHFQ), including GESICA and Wen.^30,35^ Absolute improvements in MLHFQ scores ranged from approximately 8 to 30 points favouring intervention arms, with the largest effect observed in exercise-integrated telemonitoring, where Wen *et al*. reported a ∼30 point reduction compared with ∼8–10 points in usual care.^30^ Four studies reported functional endpoints. Tele-rehabilitation significantly increased 6 min walk distance in Wen’s trial (456.9 m vs. 402.3 m in controls), and similar improvements appeared in Suthipong.^30,32^ The New York Heart Association (NYHA) class improved in multiple trials, including de Souza’s and Pan’s, although definitions and timing varied, limiting comparability.^26,28^ Self-management outcomes consistently favoured digital interventions, including substantial gains in HF self-care behaviour scores,^26^ better medication adherence,^24^ and higher health literacy scores in tele-monitored rehab.^30^ These findings were directionally consistent despite heterogeneity in scales. Several Chinese studies reported improvements in cardiac structure and function relative to usual care, including greater increases in left ventricular ejection fraction (4–6 percentage points) and stroke volume (9–10 mL), along with larger reductions in left ventricular end diastolic diameter and left ventricular end systolic diameter (2–5 mm) in intervention arms compared with controls.^27,30^ Heart rate control also improved with telemonitoring.^30^ Emergency rooom visits decreased in telemonitored interventions such as in de Souza (20.5% vs. 27.7% in controls), with similar directional findings in Domingues and Somsiri.^23,26,31^

### Sensitivity analyses

We conducted several subgroup and sensitivity analyses to evaluate the robustness of the primary findings. Excluding studies with a high risk of bias^23,33,34,36^ or those with follow-up duration <6 months^27–29,31^ did not affect estimates for hospitalization or mortality outcomes.

Reductions in all-cause hospitalization remained statistically significant across sensitivity models, with RRs ranging from approximately 0.70 to 0.76 (95% CIs 0.59–0.90), and HF-related hospitalization estimates were similarly stable (RR 0.67–0.73, 95% CIs 0.59–0.85). For mortality outcomes, estimates for all-cause mortality remained directionally favourable to dHealth interventions (RR 0.84–0.89, 95% CIs 0.74–1.00), while HF-specific mortality remained nonsignificant with wide CIs (RR range 0.64–0.75, 95% CIs 0.37–1.13).

**Figure 5:**
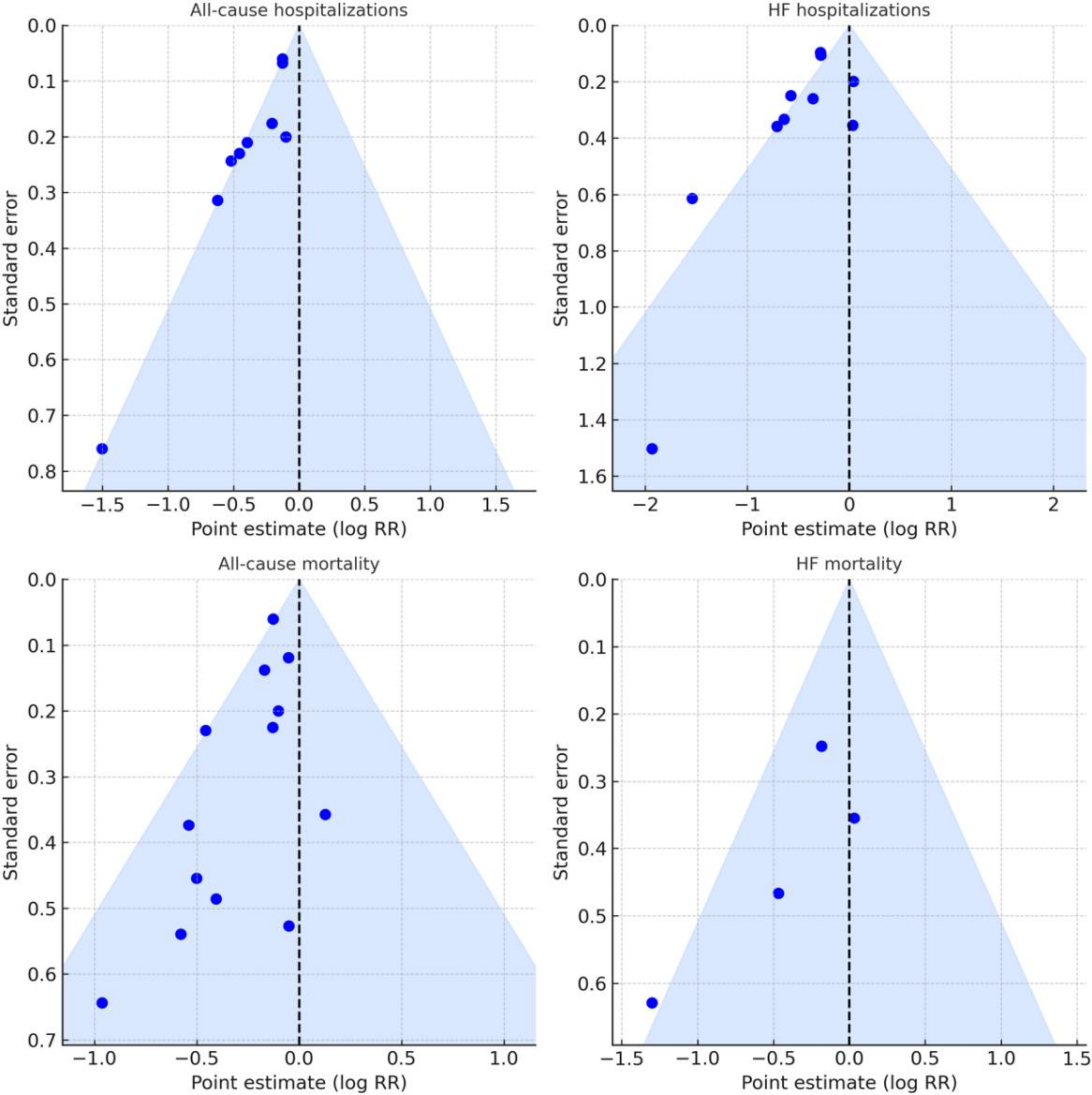
Funnel plots of included studies.

Composite-endpoint sensitivity analyses, which incorporated studies reporting combined outcomes (e.g. hospitalization or death), yielded pooled relative risks nearly identical to the primary analyses for all-cause hospitalization (RR 0.81, 95% CI 0.72–0.90) and all-cause mortality (RR 0.86, 95% CI 0.79–0.94), with modest heterogeneity (*I*^2^ 0–30%), *Figure 5*. Influence analysis using leave-one-out methods showed no single trial, including the largest multicentre programme,^35^ meaningfully changed the magnitude or direction of the pooled effects for hospitalization or mortality.

### Subgroup analyses

Subgroup analyses were performed by geographic region, intervention modality, follow-up duration, and HF severity. By geographic region, dHealth interventions were associated with reduced all-cause hospitalization across Brazil,^22–26^ other Latin American settings,^35,36^ Asia,^27–32^ and other upper-middle-income settings,^33,34^ with effect estimates consistently favouring intervention arms (overall RR range approximately 0.70–0.85 across regions, with overlapping 95% CIs). All-cause mortality estimates were similarly directionally favourable across regions (RRs <1.0), although CIs included the null value in several regional subgroups. Formal statistical testing for interaction by region was not performed; however, subgroup point estimates demonstrated overlapping CIs, and no qualitative evidence of meaningful between-region effect modification was observed.

By intervention modality, telemonitoring/telemedicine, mobile application-based interventions, SMS-based systems, and device-supported monitoring each demonstrated reductions in all-cause hospitalization, with RRs below 1.0 and overlapping CIs (RRs generally ranging from approximately 0.65 to 0.85 across modalities). Reductions in all-cause mortality were also directionally consistent across modalities (RRs <1.0), though estimates were less precise and not statistically significant. Although mobile application–based interventions, particularly in Asian studies,^27–29^ occasionally produced larger point estimates for hospitalization outcomes, these effects were imprecise and not statistically distinguishable from other delivery formats.

By follow-up duration, reductions in all-cause hospitalization were observed in both short-term (<6 months)^27–29,31^ and longer-term (≥6 months) studies,^22–26,30,32–35^ with estimates remaining statistically significant in both categories (RRs <1.0 with 95% CIs excluding 1.0). In contrast, reductions in all-cause mortality were primarily observed in studies with ≥6 months of follow-up,^22,23,25,26,35^ whereas shorter-duration studies^27–29,31^ showed wider CIs consistent with limited event accrual.

Exploratory subgroup analyses stratified studies by HF severity using reported NYHA class distributions (≥50% vs. <50% NYHA class III–IV). Reductions in hospitalization outcomes were observed in both severity strata, with pooled RRs favouring dHealth interventions in studies with predominantly higher-severity HF (≥50% NYHA class III–IV; RR approximately 0.68–0.75, 95% CIs 0.55–0.90) and in those with lower-severity populations (<50% NYHA class III–IV; RR approximately 0.72–0.80, 95% CIs 0.60–0.95), with overlapping CIs.

### Publication bias

**Figure 6:**
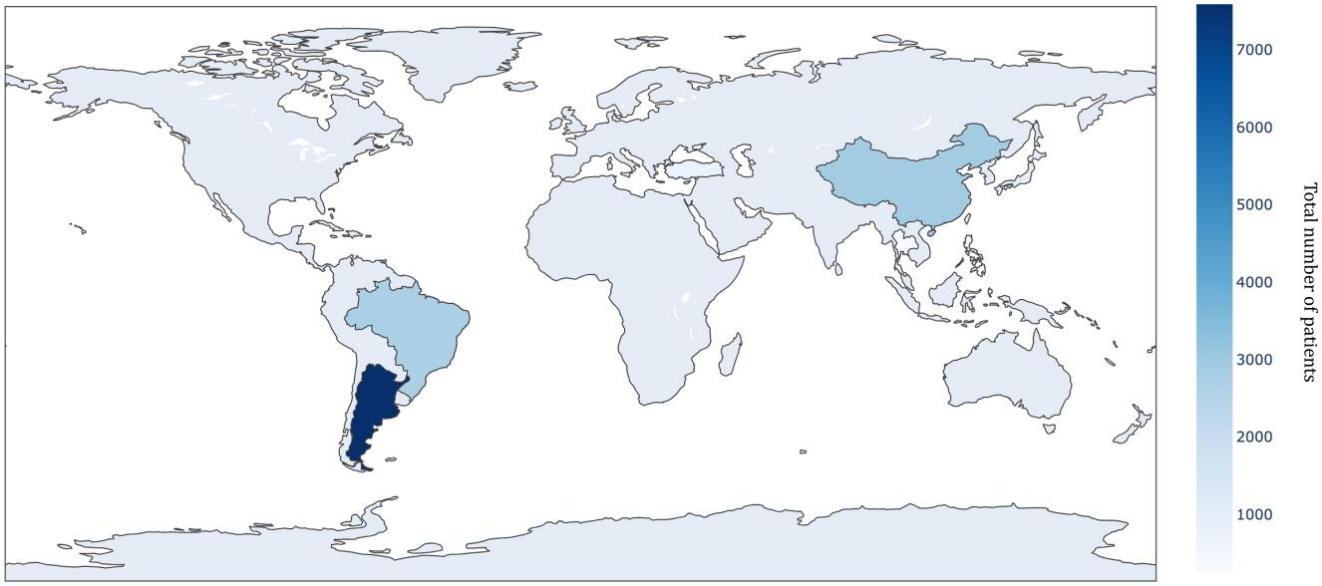
Geographic Distribution of Studies on Heart Failure in LMICs.

Funnel plots for each primary outcome were qualitatively symmetric, and Egger’s regression tests did not provide statistically significant evidence of small-study effects for all-cause hospitalizations (*P* ≈ 0.68), HF hospitalizations (*P* ≈ 0.26), all-cause mortality (*P* ≈ 0.18), or HF mortality (*P* ≈ 0.40), *Figure 6*.

## Discussion

Digital health interventions across LMIC settings were associated with meaningful reductions in all-cause and HF-specific hospitalizations, along with lower all-cause mortality. Across the 15 RCTs included in this review, dHealth tools ranging from telemonitoring/telemedicine programmes to mobile applications and device-supported monitoring improved clinical outcomes consistently. Brazilian trials^22–26^ demonstrated reductions in rehospitalization, while centralized telemonitoring in Argentina produced similar findings.^35^ Mobile application studies in China also reduced hospitalizations and improved daily monitoring behaviours.^27–29^ In Thailand, transitional telemonitoring/telemedicine and self-management support improved functional capacity and satisfaction with care.^31,32^ Mortality reductions were seen for all-cause mortality, while HF-specific mortality trended favourably but remained nonsignificant. Improvements in health literacy, medication adherence, symptom recognition, NYHA class, exercise capacity, and rehabilitation engagement were also reported. Taken together, these findings indicate that digital platforms strengthen HF management in LMICs by improving continuity of care, enabling earlier identification of decompensation, supporting structured self-management, and reducing the time between symptom onset and clinical decision-making.

Interpretation of these findings must be grounded in the realities of LMIC health systems, where barriers to routine care shape both the need for and benefits of dHealth interventions. Importantly, LMICs have experienced rapid growth in mobile phone penetration, creating new opportunities to leverage dHealth interventions for chronic disease management. At the same time, in many regions, limited post-discharge follow-up, long travel distances, and constrained specialist availability leave patients without adequate surveillance during vulnerable periods. In some settings, a single cardiologist may serve an entire district, with patients travelling hours for routine evaluations, as illustrated in Africa where a population of more than 1 billion people is served by only a few thousand cardiologists.^37^ Digital tools that allow ongoing monitoring or virtual follow-up can therefore fill critical gaps, reduce delays in care, and expand reach without requiring large-scale facility expansion.^38^ Interventions like HELEN-II, which delivered repeated nurse contacts and home assessment soon after discharge, addressed precisely these gaps.^26^ Domingues similarly demonstrated that structured telephone education and monitoring reduce readmissions in a system where traditional outpatient access is limited.^23^ In Thailand, digital transitional care supported patients after hospitalization when clinic access was inconsistent.^31,32^ In China, mobile application–based interventions allowed patients to record symptoms, vitals, and medication adherence, enabling timely clinician review and preventing progression of early decompensation.^28,29^ These mechanisms align with broader evidence showing that digital interventions in LMICs are often most effective when they compensate for systemic constraints such as geography, workforce shortages, and delays in in-person care.^39,40^ Collectively, these studies illustrate that the value of dHealth in LMICs often lies not in sophisticated technology but in solving basic access challenges.

A comparison with prior evidence highlights both similarities and important distinctions. In HIC settings, dHealth tools such as telemonitoring, mobile applications, and remote supportive care have shown mixed effects on hospitalization and mortality, in part because baseline access to specialized outpatient services is higher and routine follow-up is more consistent.^7,9^ When patients already receive frequent follow-ups, the marginal benefit of digital supplementation decreases. In LMICs, the counterfactual is often limited or inconsistent outpatient follow-up rather than comprehensive clinic-based HF management. This difference is reflected in the more consistent hospitalization reductions seen across Brazilian and Argentinian trials^23,26,35^ and the favourable results from Chinese mobile platforms.^27–29^ Global assessments have similarly suggested that dHealth provides greater relative benefit in resource-constrained systems where gaps in continuity, medication titration, and symptom surveillance are more pronounced.^41^ Evidence from remote monitoring, mHealth, and simplified triage tools in other LMIC chronic disease programmes supports this interpretation, demonstrating that digital platforms meaningfully improve outcomes when baseline care is fragmented.^42,43^ In this context, hospitalization often represents the earliest reliably captured clinical endpoint, whereas HF-specific mortality may be more difficult to ascertain due to fragmented follow-up, competing causes of death, and a rapidly evolving epidemiologic landscape in many LMICs. The observation that hospitalization outcomes improved more consistently than HF-specific mortality therefore likely reflects limitations in outcome capture and health system infrastructure rather than absence of biological effect.

Despite the promise of dHealth for HF care in LMICs, implementation must be approached with careful attention to infrastructure, socioeconomic context, and equity. These enabling conditions are not uniformly present across LMICs. Persistent barriers including intermittent electricity, low smartphone affordability, limited broadband availability, competing caregiver responsibilities, and variation in digital literacy and fluency shape whether an intervention is feasible, acceptable, or sustainable.^40,44^ Several included trials did not report baseline digital access or literacy, masking potential challenges to scalability. Even where dHealth interventions have been implemented, they vary widely in design, intensity, duration, and outcome measures, ranging from SMS reminders to app-based self-management platforms and teleconsultation services. Health system constraints also influence feasibility. Effective remote monitoring requires responsive clinical teams capable of interpreting alerts, adjusting therapy, and coordinating follow-up, yet shortages of specialized HF services and trained clinicians remain widespread in many LMICs.^37,41^ Cultural norms concerning shared phone access, privacy, and technology trust vary by region and may influence uptake. Without integration into existing health systems, dHealth tools risk creating parallel workflows that increase clinician burden rather than streamline care. Evidence from global digital scale-up experiences suggests that sustainability requires alignment with system priorities, dedicated workforce strategies, and simplified workflows.^39,40^

This systematic review has several strengths that reinforce the relevance of its findings. It is the first to synthesize RCTs of dHealth interventions for HF conducted exclusively in LMICs, incorporating more than 4500 participants across eight countries. The inclusion of non-English trials from China and Thailand broadens the evidence base beyond English-language publications, which have historically dominated dHealth literature. Intervention modalities were diverse, allowing assessment across telemonitoring, mobile applications, and SMS-based interventions. Sensitivity analyses confirmed the robustness of the main findings, with similar effect estimates when limiting to lower risk of bias studies or longer-term follow-up. Secondary outcomes such as self-care, functional capacity, and health literacy provide important mechanistic insight into how digital tools improve HF outcomes. However, limitations should be acknowledged. Many trials were modest in size, leading to imprecise mortality estimates. Reporting quality varied, and risk of bias ratings frequently reflected unclear allocation procedures, unblinded outcome assessment, or incomplete adherence reporting. Follow-up durations were generally short, and data on long-term sustainability were limited. Most trials originated from upper-middle income countries, particularly Brazil and China, limiting generalizability to low-income regions where infrastructure and resource limitations may be more severe. Socioeconomic variables, digital literacy, and cultural factors were infrequently reported, hindering equity assessments. Interventions also differed substantially in intensity and content, preventing determination of which components were most essential for improving outcomes. Although funnel plots showed no significant evidence of publication bias, unpublished negative trials cannot be excluded.

Several priorities emerge for advancing digital HF care in LMICs. Future research should expand into low-income-country regions where the burden of HF is high and dHealth access remains varied. Human-centred design approaches are needed to tailor interventions to local languages, cultural practices, literacy levels, and technology access patterns. Scalability should be a central consideration, as many effective programmes relied on intensive nurse-led interactions that may not be feasible at population scale without redesigned workflows or expanded community health worker roles. Simplified triage algorithms, automated symptom screening, and structured pathways for medication optimization may help reduce clinician workload while preserving safety, as suggested by prior mHealth and HF self-management studies.^43^ Integration of guideline-directed therapy titration into remote monitoring programmes could increase clinical impact, as suggested by improvements in medication adherence seen in Oscalices and in global dHealth analyses.^24,43^ Cost-effectiveness data remain limited but are essential for informing policy decisions in publicly financed health systems. Finally, real-world implementation studies should examine digital equity, clinician workload, and system integration to ensure that dHealth strengthens, rather than fragments, existing HF care pathways.

## Conclusion

Digital health interventions across LMICs reduced hospitalizations, lowered all-cause mortality, and improved symptom monitoring, functional status, and self-care behaviours. These findings demonstrate that relatively simple digital strategies can meaningfully strengthen HF care in settings where access to consistent outpatient management is limited. Ensuring equitable access, tailoring solutions to local contexts, and integrating digital pathways into existing health systems will be essential for maximizing impact. With continued evidence generation and thoughtful implementation, dHealth can play a key role in reducing global disparities in HF outcomes.

## Lead author biography


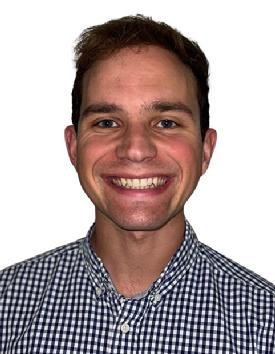
 Dr. Nicely is an Internal Medicine resident at the University of Alabama at Birmingham with a focused interest in heart failure and global health. His clinical and research work centres on digital health and implementation research, particularly in resource limited areas. Dr. Nicely is currently pursuing his masters in global health, and plans to pursue cardiology fellowship, with the goal of integrating clinical innovation, digital therapeutics, and global health equity into his future practice.

## Supplementary Material

ztag117_Supplementary_Data

## Data Availability

No new primary data were generated.
