# Supplementary material for: The impact of digital health on hospitalization and mortality in heart failure patients in low- and middle-income countries: a systematic review and meta-analysis
Source: Eur Heart J Digit Health. 2026 Jul 22;7(7):ztag117. doi: 10.1093/ehjdh/ztag117 (PMC13447851; doi:10.1093/ehjdh/ztag117)
Supplement: Supplementary file 1 [file ztag117_supplementary_data.docx]

**Supplemental Materials**

**Table S1:** PICO for systematic review

| **Criterion** | **Included** | **Excluded** |
| --- | --- | --- |
| **Population** | - LMIC populations (defined by the World Bank Classification) - Adults >18 | Non-LMIC |
| **Intervention** | - Digital Health Modality for CHF Management   - Stratify by type digital health platforms, i.e.: mobile health, artificial intelligence, wearables, text messaging, telehealth, or web-based platforms for remote monitoring of patients | No report of digital health modalities |
| **Control** | - CHF management without Digital health component (if available) |  |
| **Primary Outcomes**  (≥1 outcome of interest required) | - All-cause mortality - HF-related mortality - HF hospitalization   All-cause hospitalization |  |
| **Secondary Outcomes**  (If recorded) | - Remote symptom monitoring (NYHA class) - Emergency department visits, and clinic visits - guideline directed pharmacotherapy initiation for heart failure - lifestyle/quality of life (such as measuring symptoms and vitals, adhering to a low-sodium diet, taking prescribed medication (degree of GDMT utilization), and daily exercise) - Connection to care/referral to cardiology |  |
| **Study Type** | - Randomized trials | - Case reports - Case series - Opinion/editorial papers - Descriptive papers (White papers) - Review Articles - Observational studies with a control / reference comparator group - Observation studies with a pre/post design - Qualitative studies |
| **Language** | English and Non-english Language |  |
| **Year of Publication** | January 1, 1990-June 11, 2025 |  |

**File S2:** PubMed Search Strategy

**Databases:** PubMed, OVID: Global Health, Embase, CINAHL, Web of Science, and Cochrane Library

**Heart Failure**

("Heart Failure"[MeSH] OR "Heart Failure, Systolic"[MeSH] OR "Heart Failure, Diastolic"[MeSH] OR "Ventricular Dysfunction, Left"[MeSH] OR "Ventricular Dysfunction, Right"[MeSH] OR "Cardiomyopathies"[MeSH] OR "Cardiomegaly"[MeSH] OR "Pulmonary Edema"[MeSH] OR "Echocardiography"[MeSH] OR "Cardiac Output"[MeSH] OR "Ischemic Heart Disease"[MeSH] OR "Myocardial Ischemia"[MeSH] OR heart failure[tiab] OR congestive heart failure[tiab] OR CHF[tiab] OR cardiomyopathy[tiab] OR cardiomyopathies[tiab] OR ischemic heart disease[tiab] OR nonischemic heart disease[tiab] OR non-ischemic heart disease[tiab] OR myocardial ischemia[tiab] OR decompensated heart failure[tiab] OR decompensation[tiab] OR reduced ejection fraction[tiab] OR preserved ejection fraction[tiab] OR mid-range ejection fraction[tiab] OR ejection fraction[tiab] OR HFrEF[tiab] OR HFpEF[tiab] OR HFmrEF[tiab] OR HFrecEF[tiab] OR ventricular dilation[tiab] OR ventricular dilatation[tiab] OR dilated ventricle[tiab] OR cardiomegaly[tiab] OR shortness of breath[tiab] OR dyspnea[tiab] OR dyspnea on exertion[tiab] OR orthopnea[tiab] OR paroxysmal nocturnal dyspnea[tiab] OR PND[tiab] OR BNP[tiab] OR pro-BNP[tiab] OR NT-proBNP[tiab] OR echocardiography[tiab] OR echo[tiab] OR pulmonary edema[tiab] OR jugular venous distension[tiab] OR JVD[tiab] OR S3[tiab] OR ventricular gallop[tiab] OR lower extremity edema[tiab] OR peripheral edema[tiab] OR left-sided heart failure[tiab] OR right-sided heart failure[tiab] OR systolic heart failure[tiab] OR diastolic heart failure[tiab] OR acute heart failure[tiab] OR chronic heart failure[tiab] OR cardiac output[tiab] OR cardiac dysfunction[tiab] OR volume overload[tiab] OR exercise intolerance[tiab])

**Digital Health**

("Telemedicine"[MeSH] OR "Mobile Applications"[MeSH] OR "Cell Phones"[MeSH] OR "Smartphone"[MeSH] OR "Medical Informatics"[MeSH] OR "Medical Informatics Applications"[MeSH] OR "Remote Sensing Technology"[MeSH] OR "Wireless Technology"[MeSH] OR "Telemetry"[MeSH] OR "Electronic Mail"[MeSH] OR "Social Media"[MeSH] OR "Internet"[MeSH] OR "Videoconferencing"[MeSH] OR "Ambulatory Blood Pressure Monitoring"[MeSH] OR "Ambulatory Electrocardiography"[MeSH] OR digital technology[tiab] OR digital health[tiab] OR digital intervention*[tiab] OR digital application*[tiab] OR digital app*[tiab] OR mobile health[tiab] OR mhealth[tiab] OR m-health[tiab] OR mobile phone*[tiab] OR mobile device*[tiab] OR cell phone*[tiab] OR cellular phone*[tiab] OR smart phone*[tiab] OR smartphone*[tiab] OR telecommunication*[tiab] OR SMS[tiab] OR text messag*[tiab] OR texting[tiab] OR telemedicine[tiab] OR telehealth[tiab] OR digital[tiab] OR online[tiab] OR web[tiab] OR web-based[tiab] OR website-based[tiab] OR internet-based[tiab] OR social media[tiab] OR mobile application*[tiab] OR mobile app*[tiab] OR apps[tiab] OR wearable*[tiab] OR fitness tracker*[tiab] OR activity tracker*[tiab] OR digital vending machine*[tiab] OR monitoring device*[tiab] OR remote monitoring[tiab] OR remote sensor*[tiab] OR remote sensing technolog*[tiab] OR Bluetooth[tiab] OR wireless technolog*[tiab] OR telemetry[tiab] OR radio[tiab] OR television[tiab] OR videoconferencing[tiab] OR video conferencing[tiab] OR medical informatics[tiab] OR informatics[tiab] OR electronic mail[tiab] OR email[tiab] OR e-mail[tiab] OR ambulatory blood pressure monitoring[tiab] OR ABPM[tiab] OR ambulatory electrocardiography[tiab] OR Holter[tiab])

**LMICs:**

("developing countries"[MeSH Terms] OR "africa"[MeSH Terms] OR "asia"[MeSH Terms:noexp] OR "asia, central"[MeSH Terms] OR "asia, southeastern"[MeSH Terms] OR "asia, western"[MeSH Terms] OR "caribbean region"[MeSH Terms] OR "south america"[MeSH Terms] OR "latin america"[MeSH Terms] OR "central america"[MeSH Terms] OR "afghanistan"[MeSH Terms] OR "albania"[MeSH Terms] OR "algeria"[MeSH Terms] OR "american samoa"[MeSH Terms] OR "angola"[MeSH Terms] OR "antigua and barbuda"[MeSH Terms] OR "argentina"[MeSH Terms] OR "armenia"[MeSH Terms] OR "azerbaijan"[MeSH Terms] OR "bahrain"[MeSH Terms] OR "bangladesh"[MeSH Terms] OR "barbados"[MeSH Terms] OR "benin"[MeSH Terms] OR "republic of belarus"[MeSH Terms] OR "belize"[MeSH Terms] OR "bhutan"[MeSH Terms] OR "bolivia"[MeSH Terms] OR "bosnia and herzegovina"[MeSH Terms] OR "botswana"[MeSH Terms] OR "brazil"[MeSH Terms] OR "bulgaria"[MeSH Terms] OR "burkina faso"[MeSH Terms] OR "burundi"[MeSH Terms] OR "cambodia"[MeSH Terms] OR "cameroon"[MeSH Terms] OR "cabo verde"[MeSH Terms] OR "central african republic"[MeSH Terms] OR "chad"[MeSH Terms] OR "chile"[MeSH Terms] OR "china"[MeSH Terms] OR "colombia"[MeSH Terms] OR "comoros"[MeSH Terms] OR "congo"[MeSH Terms] OR "costa rica"[MeSH Terms] OR "cote d ivoire"[MeSH Terms] OR "croatia"[MeSH Terms] OR "cuba"[MeSH Terms] OR "cyprus"[MeSH Terms] OR "czechoslovakia"[MeSH Terms] OR "czech republic"[MeSH Terms] OR "slovakia"[MeSH Terms] OR "djibouti"[MeSH Terms] OR "democratic republic of the congo"[MeSH Terms] OR "dominica"[MeSH Terms] OR "dominican republic"[MeSH Terms] OR "timor leste"[MeSH Terms] OR "ecuador"[MeSH Terms] OR "egypt"[MeSH Terms] OR "el salvador"[MeSH Terms] OR "eritrea"[MeSH Terms] OR "estonia"[MeSH Terms] OR "ethiopia"[MeSH Terms] OR "fiji"[MeSH Terms] OR "gabon"[MeSH Terms] OR "gambia"[MeSH Terms] OR "georgia republic"[MeSH Terms] OR "ghana"[MeSH Terms] OR "greece"[MeSH Terms] OR "grenada"[MeSH Terms] OR "guatemala"[MeSH Terms] OR "guinea"[MeSH Terms] OR "guinea bissau"[MeSH Terms] OR "guam"[MeSH Terms] OR "guyana"[MeSH Terms] OR "haiti"[MeSH Terms] OR "honduras"[MeSH Terms] OR "hungary"[MeSH Terms] OR "india"[MeSH Terms] OR "indonesia"[MeSH Terms] OR "iran"[MeSH Terms] OR "iraq"[MeSH Terms] OR "jamaica"[MeSH Terms] OR "jordan"[MeSH Terms] OR "kazakhstan"[MeSH Terms] OR "kenya"[MeSH Terms] OR "korea"[MeSH Terms] OR "kosovo"[MeSH Terms] OR "kyrgyzstan"[MeSH Terms] OR "laos"[MeSH Terms] OR "latvia"[MeSH Terms] OR "lebanon"[MeSH Terms] OR "lesotho"[MeSH Terms] OR "liberia"[MeSH Terms] OR "libya"[MeSH Terms] OR "lithuania"[MeSH Terms] OR "republic of north macedonia"[MeSH Terms] OR "madagascar"[MeSH Terms] OR "malaysia"[MeSH Terms] OR "malawi"[MeSH Terms] OR "mali"[MeSH Terms] OR "malta"[MeSH Terms] OR "mauritania"[MeSH Terms] OR "mauritius"[MeSH Terms] OR "mexico"[MeSH Terms] OR "micronesia"[MeSH Terms] OR "middle east"[MeSH Terms] OR "moldova"[MeSH Terms] OR "mongolia"[MeSH Terms] OR "montenegro"[MeSH Terms] OR "morocco"[MeSH Terms] OR "mozambique"[MeSH Terms] OR "myanmar"[MeSH Terms] OR "namibia"[MeSH Terms] OR "nepal"[MeSH Terms] OR "netherlands antilles"[MeSH Terms] OR "new caledonia"[MeSH Terms] OR "nicaragua"[MeSH Terms] OR "niger"[MeSH Terms] OR "nigeria"[MeSH Terms] OR "oman"[MeSH Terms] OR "pakistan"[MeSH Terms] OR "palau"[MeSH Terms] OR "panama"[MeSH Terms] OR "papua new guinea"[MeSH Terms] OR "paraguay"[MeSH Terms] OR "peru"[MeSH Terms] OR "philippines"[MeSH Terms] OR "poland"[MeSH Terms] OR "portugal"[MeSH Terms] OR "puerto rico"[MeSH Terms] OR "romania"[MeSH Terms] OR "russia"[MeSH Terms] OR "russia pre 1917"[MeSH Terms] OR "rwanda"[MeSH Terms] OR "saint kitts and nevis"[MeSH Terms] OR "saint lucia"[MeSH Terms] OR "saint vincent and the grenadines"[MeSH Terms] OR "samoa"[MeSH Terms] OR "saudi arabia"[MeSH Terms] OR "senegal"[MeSH Terms] OR "serbia"[MeSH Terms] OR "montenegro"[MeSH Terms] OR "seychelles"[MeSH Terms] OR "sierra leone"[MeSH Terms] OR "slovenia"[MeSH Terms] OR "sri lanka"[MeSH Terms] OR "somalia"[MeSH Terms] OR "south africa"[MeSH Terms] OR "sudan"[MeSH Terms] OR "suriname"[MeSH Terms] OR "eswatini"[MeSH Terms] OR "syria"[MeSH Terms] OR "tajikistan"[MeSH Terms] OR "tanzania"[MeSH Terms] OR "thailand"[MeSH Terms] OR "togo"[MeSH Terms] OR "tonga"[MeSH Terms] OR "trinidad and tobago"[MeSH Terms] OR "tunisia"[MeSH Terms] OR "turkey"[MeSH Terms] OR "turkmenistan"[MeSH Terms] OR "uganda"[MeSH Terms] OR "ukraine"[MeSH Terms] OR "uruguay"[MeSH Terms] OR "ussr"[MeSH Terms] OR "uzbekistan"[MeSH Terms] OR "vanuatu"[MeSH Terms] OR "venezuela"[MeSH Terms] OR "vietnam"[MeSH Terms] OR "yemen"[MeSH Terms] OR "yugoslavia"[MeSH Terms] OR "zambia"[MeSH Terms] OR "zimbabwe"[MeSH Terms] OR "developing country"[Text Word] OR "developing countries"[Text Word] OR "developing nation"[Text Word] OR "developing nations"[Text Word] OR "developing population"[Text Word] OR "developing populations"[Text Word] OR "developing world"[Text Word] OR "less developed country"[Text Word] OR "less developed countries"[Text Word] OR "less developed nation"[Text Word] OR "less developed nations"[Text Word] OR "less developed world"[Text Word] OR "lesser developed countries"[Text Word] OR "lesser developed nations"[Text Word] OR "least developed country"[Text Word] OR "least developed countries"[Text Word] OR "least developed nations"[Text Word] OR "under developed country"[Text Word] OR "under developed countries"[Text Word] OR "under developed nations"[Text Word] OR "under developed world"[Text Word] OR "underdeveloped country"[Text Word] OR "underdeveloped countries"[Text Word] OR "underdeveloped nation"[Text Word] OR "underdeveloped nations"[Text Word] OR "underdeveloped population"[Text Word] OR "underdeveloped populations"[Text Word] OR "underdeveloped world"[Text Word] OR "middle income country"[Text Word] OR "middle income countries"[Text Word] OR "middle income nation"[Text Word] OR "middle income nations"[Text Word] OR "middle income population"[Text Word] OR "middle income populations"[Text Word] OR "low income country"[Text Word] OR "low income countries"[Text Word] OR "low income nation"[Text Word] OR "low income nations"[Text Word] OR "low income population"[Text Word] OR "low income populations"[Text Word] OR "lower income country"[Text Word] OR "lower income countries"[Text Word] OR "lower income nations"[Text Word] OR "lower income population"[Text Word] OR "lower income populations"[Text Word] OR "underserved countries"[Text Word] OR "underserved nations"[Text Word] OR "underserved population"[Text Word] OR "underserved populations"[Text Word] OR "under served population"[Text Word] OR "under served populations"[Text Word] OR "deprived countries"[Text Word] OR "deprived population"[Text Word] OR "deprived populations"[Text Word] OR "poor country"[Text Word] OR "poor countries"[Text Word] OR "poor nation"[Text Word] OR "poor nations"[Text Word] OR "poor population"[Text Word] OR "poor populations"[Text Word] OR "poor world"[Text Word] OR "poorer countries"[Text Word] OR "poorer nations"[Text Word] OR "poorer population"[Text Word] OR "poorer populations"[Text Word] OR "developing economy"[Text Word] OR "developing economies"[Text Word] OR "less developed economy"[Text Word] OR "less developed economies"[Text Word] OR "underdeveloped economies"[Text Word] OR "middle income economy"[Text Word] OR "middle income economies"[Text Word] OR "low income economy"[Text Word] OR "low income economies"[Text Word] OR "lower income economies"[Text Word] OR "low gdp"[Text Word] OR "low gnp"[Text Word] OR "low gross domestic"[Text Word] OR "low gross national"[Text Word] OR "lower gdp"[Text Word] OR "lower gross domestic"[Text Word] OR "lmic"[Text Word] OR "lmics"[Text Word] OR "third world"[Text Word] OR "lami country"[Text Word] OR "lami countries"[Text Word] OR "transitional country"[Text Word] OR "transitional countries"[Text Word] OR "africa"[Text Word] OR "asia"[Text Word] OR "west indies"[Text Word] OR "south america"[Text Word] OR "latin america"[Text Word] OR "central america"[Text Word] OR "afghanistan"[Text Word] OR "albania"[Text Word] OR "algeria"[Text Word] OR "angola"[Text Word] OR "antigua"[Text Word] OR "barbuda"[Text Word] OR "argentina"[Text Word] OR "armenia"[Text Word] OR "armenian"[Text Word] OR "aruba"[Text Word] OR "azerbaijan"[Text Word] OR "bahrain"[Text Word] OR "bangladesh"[Text Word] OR "barbados"[Text Word] OR "benin"[Text Word] OR "byelarus"[Text Word] OR "byelorussian"[Text Word] OR "belarus"[Text Word] OR "belorussian"[Text Word] OR "belorussia"[Text Word] OR "belize"[Text Word] OR "bhutan"[Text Word] OR "bolivia"[Text Word] OR "bosnia"[Text Word] OR "herzegovina"[Text Word] OR "hercegovina"[Text Word] OR "botswana"[Text Word] OR "brasil"[Text Word] OR "brazil"[Text Word] OR "bulgaria"[Text Word] OR "burkina faso"[Text Word] OR "burkina fasso"[Text Word] OR "upper volta"[Text Word] OR "burundi"[Text Word] OR "urundi"[Text Word] OR "cambodia"[Text Word] OR "khmer republic"[Text Word] OR "kampuchea"[Text Word] OR "cameroon"[Text Word] OR "cameroons"[Text Word] OR "cameron"[Text Word] OR "cape verde"[Text Word] OR "central african republic"[Text Word] OR "chad"[Text Word] OR "chile"[Text Word] OR "china"[Text Word] OR "colombia"[Text Word] OR "comoros"[Text Word] OR "comoro islands"[Text Word] OR "comores"[Text Word] OR "mayotte"[Text Word] OR "congo"[Text Word] OR "zaire"[Text Word] OR "costa rica"[Text Word] OR "cote d ivoire"[Text Word] OR "ivory coast"[Text Word] OR "croatia"[Text Word] OR "cuba"[Text Word] OR "cyprus"[Text Word] OR "czechoslovakia"[Text Word] OR "czech republic"[Text Word] OR "slovakia"[Text Word] OR "slovak republic"[Text Word] OR "djibouti"[Text Word] OR "french somaliland"[Text Word] OR "dominica"[Text Word] OR "dominican republic"[Text Word] OR "east timor"[Text Word] OR "timor leste"[Text Word] OR "ecuador"[Text Word] OR "egypt"[Text Word] OR "united arab republic"[Text Word] OR "el salvador"[Text Word] OR "eritrea"[Text Word] OR "estonia"[Text Word] OR "ethiopia"[Text Word] OR "fiji"[Text Word] OR "gabon"[Text Word] OR "gabonese republic"[Text Word] OR "gambia"[Text Word] OR "gaza"[Text Word] OR "georgia republic"[Text Word] OR "georgian republic"[Text Word] OR "ghana"[Text Word] OR "gold coast"[Text Word] OR "greece"[Text Word] OR "grenada"[Text Word] OR "guatemala"[Text Word] OR "guinea"[Text Word] OR "guam"[Text Word] OR "guiana"[Text Word] OR "guyana"[Text Word] OR "haiti"[Text Word] OR "honduras"[Text Word] OR "hungary"[Text Word] OR "india"[Text Word] OR "maldives"[Text Word] OR "indonesia"[Text Word] OR "iran"[Text Word] OR "iraq"[Text Word] OR "isle of man"[Text Word] OR "jamaica"[Text Word] OR "jordan"[Text Word] OR "kazakhstan"[Text Word] OR "kazakh"[Text Word] OR "kenya"[Text Word] OR "kiribati"[Text Word] OR "korea"[Text Word] OR "kosovo"[Text Word] OR "kyrgyzstan"[Text Word] OR "kirghizia"[Text Word] OR "kyrgyz republic"[Text Word] OR "kirghiz"[Text Word] OR "kirgizstan"[Text Word] OR "lao pdr"[Text Word] OR "laos"[Text Word] OR "latvia"[Text Word] OR "lebanon"[Text Word] OR "lesotho"[Text Word] OR "basutoland"[Text Word] OR "liberia"[Text Word] OR "libya"[Text Word] OR "lithuania"[Text Word] OR "macedonia"[Text Word] OR "madagascar"[Text Word] OR "malagasy republic"[Text Word] OR "malaysia"[Text Word] OR "malaya"[Text Word] OR "malay"[Text Word] OR "sabah"[Text Word] OR "sarawak"[Text Word] OR "malawi"[Text Word] OR "nyasaland"[Text Word] OR "mali"[Text Word] OR "malta"[Text Word] OR "marshall islands"[Text Word] OR "mauritania"[Text Word] OR "mauritius"[Text Word] OR "agalega islands"[Text Word] OR "mexico"[Text Word] OR "micronesia"[Text Word] OR "moldova"[Text Word] OR "moldovia"[Text Word] OR "moldovian"[Text Word] OR "mongolia"[Text Word] OR "montenegro"[Text Word] OR "morocco"[Text Word] OR "ifni"[Text Word] OR "mozambique"[Text Word] OR "myanmar"[Text Word] OR "myanma"[Text Word] OR "burma"[Text Word] OR "namibia"[Text Word] OR "nepal"[Text Word] OR "netherlands antilles"[Text Word] OR "new caledonia"[Text Word] OR "nicaragua"[Text Word] OR "niger"[Text Word] OR "nigeria"[Text Word] OR "northern mariana islands"[Text Word] OR "oman"[Text Word] OR "muscat"[Text Word] OR "pakistan"[Text Word] OR "palau"[Text Word] OR "palestine"[Text Word] OR "panama"[Text Word] OR "paraguay"[Text Word] OR "peru"[Text Word] OR "philippines"[Text Word] OR "philipines"[Text Word] OR "phillipines"[Text Word] OR "phillippines"[Text Word] OR "poland"[Text Word] OR "portugal"[Text Word] OR "puerto rico"[Text Word] OR "rhodesia"[Text Word] OR "romania"[Text Word] OR "rumania"[Text Word] OR "roumania"[Text Word] OR "russia"[Text Word] OR "russian"[Text Word] OR "rwanda"[Text Word] OR "ruanda"[Text Word] OR "saint kitts"[Text Word] OR "st kitts"[Text Word] OR "nevis"[Text Word] OR "saint lucia"[Text Word] OR "st lucia"[Text Word] OR "saint vincent"[Text Word] OR "st vincent"[Text Word] OR "grenadines"[Text Word] OR "samoa"[Text Word] OR "samoan islands"[Text Word] OR "sao tome"[Text Word] OR "saudi arabia"[Text Word] OR "senegal"[Text Word] OR "serbia"[Text Word] OR "montenegro"[Text Word] OR "seychelles"[Text Word] OR "sierra leone"[Text Word] OR "slovenia"[Text Word] OR "sri lanka"[Text Word] OR "ceylon"[Text Word] OR "solomon islands"[Text Word] OR "somalia"[Text Word] OR "sudan"[Text Word] OR "suriname"[Text Word] OR "surinam"[Text Word] OR "swaziland"[Text Word] OR "syria"[Text Word] OR "tajikistan"[Text Word] OR "tadzhikistan"[Text Word] OR "tadjikistan"[Text Word] OR "tadzhik"[Text Word] OR "tanzania"[Text Word] OR "thailand"[Text Word] OR "togo"[Text Word] OR "togolese republic"[Text Word] OR "tonga"[Text Word] OR "trinidad"[Text Word] OR "tobago"[Text Word] OR "tunisia"[Text Word] OR "turkey"[Text Word] OR "turkmenistan"[Text Word] OR "turkmen"[Text Word] OR "uganda"[Text Word] OR "ukraine"[Text Word] OR "uruguay"[Text Word] OR "ussr"[Text Word] OR "soviet union"[Text Word] OR "union of soviet socialist republics"[Text Word] OR "uzbekistan"[Text Word] OR "vanuatu"[Text Word] OR "new hebrides"[Text Word] OR "venezuela"[Text Word] OR "vietnam"[Text Word] OR "viet nam"[Text Word] OR "west bank"[Text Word] OR "yemen"[Text Word] OR "yugoslavia"[Text Word] OR "zambia"[Text Word] OR "zimbabwe"[Text Word])

# **Table S3:** Composite Outcomes

| Author (Year) | Composite Definition | Outcome |
| --- | --- | --- |
| Domingues 2010 | CV hospitalization or death due to HF | HF hospitalization, HF mortality |
| Domingues 2010 | All-cause rehospitalization or all-cause death | All-cause hospitalization, All-cause mortality |
| GESICA 2005 | CV admission and/or all-cause mortality | HF hospitalization |
| GESICA 2005 | All-cause admission and/or all-cause mortality | All-cause hospitalization |
| Ribeiro 2025 | All-cause death + hospitalization | All-cause hospitalization, All-cause mortality |
| Isaeva 2024 | CV death, readmission due to CHF decompensation, and ED/ambulance visits | HF hospitalization, HF mortality |
| Achury-SaldaNA 2024 | Combined CV death + HF hospitalization context | HF mortality |
| Chen 2018 | HF-related admission or death | HF mortality, HF hospitalization |
| Bocchi 2008 | Mortality likely combined with hospitalization | All-cause mortality |
| de Souza 2014 | HF hospitalization + possible ED visits | HF hospitalization |
| Suthipong 2018 | Rehospitalization or death due to all causes | All-cause hospitalization |
|  |  |  |

| Inclusion criteria | Exclusion criteria |
| --- | --- |
| i. mHealth is addressed as a primary issue | i. If it is about electronic medical records and data management |
| ii. mHealth implementation and feedback are discussed in the paper | ii. If it is about general IT and ICT technology and data collection |
| iii. The mHealth approach is towards the human domain | iii. The mHealth approach is not towards the human domain (i.e., plants, livestock, etc.) |

**Table S4:** Inclusion and Exclusion dHealth Criteria

**Table S5:** Study Specific Intervention Descriptions

| **Study Country** | **Brief Intervention Description** |
| --- | --- |
| GESICA (Argentina) | Nurses trained in the management of patients with chronic heart failure did frequent telephone follow-up from the telephone intervention center. The intervention was based on five main objectives: adherence to diet, adherence to drug treatment, monitoring of symptoms (especially progression of dyspnea and fatigue), control of signs of hydro saline retention (daily weight and edema), and daily physical activity. To achieve these objectives, nurses followed a predetermined questionnaire and standardized intervention procedures. Nurses had special software with which they recorded data on every call. |
| Bocchi (Brazil) | A disease management program (DMP) was designed consisting of a long-term repetitive multidisciplinary education program and telephone monitoring. The DMP focused on  intervention through education for patients and caregivers; medication management with optimized therapy based on guidelines and remote monitoring; delivery personnel with nurses, cardiologists, pharmacists, social workers, dietitians, dentists, and psychologists; face-to-face individual/group communication; and telephone in-person communication. Telephone calls were led by nurses trained in HF management with content derived from a predetermined questionnaire.  Through this intervention, they could do things like adjust diuretics or recommend emergency room visits. When nurses detected noncompliance or worsening of the clinical condition, an unscheduled visit to the attending cardiologist could be proposed. |
| Domingues (Brazil) | Telephone monitoring after discharge, aiming at clarifying and reinforcing instructions received during hospitalization and monitoring signs and symptoms of decompensation and investigating possible visits to the emergency care unit and rehospitalizations. The telephone call was standardized and followed the study protocol. The nurse did not interfere with or change the patients’ pharmacological treatment but recommended seeing the doctor or going to the emergency care unit when there were signs of HF decompensation. |
| Oscalices (Brazil) | Telephone call focusing on the patient’s knowledge about the disease and  treatment; need of maintenance of water, caloric and sodium restriction; performance of physical activity, if prescribed; need to control weight; knowledge about the signs and symptoms of HF decompensation; and importance of outpatient follow-up. |
| Ribeiro (Brazil) | The intervention consisted of a structured telephone support (STS) program, a self-care educational remote program (consisting of twice a week unidirectional semi-personalized text message, and a 2-way channel for questions through text messages to the case manager, during working hours. The case managers were linked to a cardiologist who was activated for teleconsultations, according to predefined clinical decision trees based on clinical status and alarm signs. STS evaluated the following: the daily monitoring of weight, blood pressure, and heart rate collected by patients, to assess decompensation signs and symptoms, to address treatment adherence barriers, and to promote self-care education, including adjustments of diuretic dosing. |
| Souza-HELEN II (Brazil) | Four home visits with four reinforcement telephone calls, at 10 days post discharge, 30 days, 60 days, and 120 days. The calls reinforced recommendations given during HVs, check the use of prescribed medications, and clarify common doubts regarding the disease and its treatment. |
| Çavuşoğlu (Turkey) | During discharge, patients were educated by a cardiologist and a nurse on HF management and a HF education booklet was provided. Patients were followed for 6 months after randomization and were contacted by telephone at 1- and 3-month and by telephone and/or an in-hospital visit after  6 months by a study cardiologist or study nurse to collect clinical data and to go over the HF education material. During the telephone follow up, correction of doses or regimen of medication were made by a cardiologist or a nurse under the supervision of a cardiologist. Patients were invited to come to the hospital if needed. |
| Chen (China) | Patients in the short messages service (SMS) group as well as their caregivers received standardized messages from text messaging platform operated by research nurses. There were two kinds of messages‚ educational SMS and reminder SMS. The educational SMSs were condensed messages with knowledge of HF (e.g. symptoms of HF decompensation), while the reminder SMSs were brief messages that prompted patients to do things (e.g. taking medicine or weighing). All educational messages were sent within the first 10 days after discharge, and then the reminder messages were repeated weekly for 1 month. The messages were scheduled to send out automatically. Patients were informed not to reply to the messages. Patients in the structured telephone service (STS) group received one structured phone call from research nurses within 30 days after discharge, which was made following pre-written structured materials. The nurses were trained with simulation before study. Patients were allowed to call back the nurses during work time for consultation. |
| Pan (China) | Mobile phone heart failure management application. Patients could log body weight, HR data, BP data |
| Qiu (China) | A "heart management" system consisting of patient version mobile terminal, medical version mobile terminal, and computer version management terminal. |
| Gaoquin (China) | Remote cardiac (EKG) monitoring during cardiac rehab with device provided by Wuhan Mingde Biotechnology (iHolter). It included a special mobile phone, early warning devices, data cables, and lead wires. |
| Somsiri (Thailand) | A mobile app (Tid Tam Jai--meaning follow the heart) was downloaded onto participants phones. The 6-week home telemonitoring phase began within 24 hours following discharge through the mobile app and telephone calls. The app was used as a channel for health communication and education between participants and healthcare teams. It was also used to record self-monitoring data: pulse, blood pressure, weight, and HF symptoms. The app also included an alert system, private messaging, and bulletin board. The alert system notified the healthcare team via email when participants failed to comply with requirements and/or when the data showed health risks. The private messaging resource was used for HF-specific knowledge‚ and the bulletin board provided general HF-related health information for all participants concomitantly. The app also included an emergency assistant for emergency telephone numbers. |
| Suthipong (Thailand) | The Heart Failure Aide Application (HFAA) is an Android app established with help from the education program for patients  with HF in Thailand who supports patients with HF by helping them care for themselves at home. The app is composed of education on HF disease, diuretic computing, fluid intake computing, hospital warning signs, and emergency interaction. |
| Isaeva (Russia) | A telephone survey and remote software  monitoring was conducted by a nurse using a predesigned algorithm, with further strategies based on patient responses. Patients received a daily notification about the need to enter their parameters (BP, HR, and body weight)  into the system for clinical disease monitoring. Well-being and adherence questionnaires were sent every 3 days. In addition, all patients were instructed in advance (at the randomization visit) to perform daily self-monitoring of BP, HR, and body weight. |
| Achury-Saldana (Colombia) | An app called ControlVit, was designed for this intervention. It had two components: a Web-enabled application for health professionals and a mobile application to enter data on the patients' health status, which allows monitoring in real time in order to detect complications in an early manner. ControlVit was installed on the patients' phone and allowed them to enter their measurements daily (weight, arterial pressure, heart rate, and symptoms questionnaire). It also contained educational information oriented to self-care, including notifications for reminders and informational capsules. The patients were provided with a scale and a digital sphygmomanometer, calibrated and certified according to national standards. The questionnaire consisted of eight yes or no items devised by the authors based on existing guidelines: (1) My feet are more swollen than yesterday; (2) I feel more fatigued or breathless than yesterday; (3) I had a bad night due to breathlessness; (4) I had to add more pillows; (5) I had to sleep sitting up due to breathlessness; (6) I felt more dizzy or weak than yesterday; (7) I had chest pain; (8) Overall, I feel worse today than yesterday. The nurse verified each alert through a phone call and intervened through algorithms or actions plans incorporated into the Web application, oriented to decision-making, endorsed by the clinical team, and based on existing clinical practice guidelines. |

**Table S6**

A list of excluded studies and the rationale for their exclusion has been digitally submitted with this revision

**Supplement S7: Deviations from PROSPERO-Registered Protocol**

**Registered Protocol:** CRD420251079715

To ensure reproducibility and external validity, we provide a detailed comparison between the originally registered PROSPERO protocol and the final methods used in this review. The protocol was initially designed as a broad umbrella review of dHealth for heart failure across multiple outcome domains and study designs. During the review, the project was refined into three complementary analyses addressing distinct research aims. The present manuscript corresponds to the analysis of objective clinical outcomes (hospitalization and mortality).

The following deviations from the original protocol were made:

**1. Restriction to Randomized Controlled Trials (RCTs)**

Protocol: Inclusion of randomized and non-randomized studies.
Final review: Inclusion restricted to randomized controlled trials.

This change was made to improve internal validity. Meta-analysis using risk ratios requires comparable study designs, and non-randomized studies would introduce substantial heterogeneity and bias into this review. Restricting to RCTs only ensured more reliable pooled estimates for our relatively small (n=15) number of included studies.

**2. Restriction of Outcomes to Event-Based Clinical Endpoints**

Protocol: Broad outcomes including diagnosis, symptom monitoring, quality of life, and healthcare utilization.
Final review: Outcomes limited to hospitalization and mortality only.

This analysis focused on defined clinical events which allowed for consistent extraction across studies. These outcomes were appropriate for quantitative synthesis using risk ratios, and other outcomes were too heterogeneous for pooled meta-analysis. Complementary reviews without meta-analysis will focus on these additional endpoints mentioned in the published protocol.

**3. Inclusion of Secondary Outcomes for Narrative Synthesis**

Protocol: Secondary outcomes were broadly defined but not specified for this analysis.
Final review: Secondary outcomes were included narratively

The secondary outcomes described in this review provided additional clinical context and were not included in the meta-analysis. While their definitions varied across studies, their brief inclusion as qualitative synthesis added to the descriptive outcomes of this work.

**4. Exclusion of Studies Without Discrete Event-Based Outcomes**

Protocol: Inclusion of studies evaluating diverse digital health applications.
Final review: Exclusion of studies without extractable clinical event data.

Some studies reported composite or biomarker-driven endpoints, while other studies reported only patient-reported outcomes. Therefore, these could not be converted into risk ratios and including them would reduce analytic consistency. These studies were retained for separate analyses aligned with other aims intended to be published in complementary work.

**5. Refinement of Intervention Classification Framework**

Protocol: Broad classification of multiple digital health modalities.
Final review: Interventions grouped into four standardized categories.

Only modalities present in the included studies were retained which improved consistency across the dataset, reduced ambiguity in classification, and ultimately improves reproducibility.
